# Wrinkled CNTs@PLLA Composite Membranes for Enhanced Separation Performance

**DOI:** 10.3390/membranes12030278

**Published:** 2022-02-28

**Authors:** Jinyan Xu, Bajin Chen, Lu Yin, Liang Zhang, Yongjin Li, Jichun You

**Affiliations:** 1 Key Laboratory of Organosilicon Chemistry and Material Technology, Ministry of Education, College of Material, Chemistry and Chemical Engineering, Hangzhou Normal University, Hangzhou 311121, China; xjy5282022@163.com (J.X.); 15967102282@163.com (L.Y.); layzhang@foxmail.com (L.Z.); yongjin-li@hznu.edu.cn (Y.L.); 2 Transfar Zhilian Co., Ltd., Xiaoshan, Hangzhou 311215, China; 4437@etransfar.com

**Keywords:** separation, wrinkle, shape memory effect, CNTs, trade-off effect

## Abstract

To break the trade-off effect between permeability and selectivity in separation, wrinkled carbon nanotubes@polylactic acid (CNTs@PLLA) composite membranes were successfully fabricated in this work. On pre-deformed PLLA membranes, CNTs were loaded by filtrating their suspension, followed by releasing the PLLA upon heating based on its shape memory effect. The asynchronous deformations of CNTs and PLLA layers produced wrinkled CNTs@PLLA composite membranes. Relative to the reference without wrinkles, the attained wrinkled composite membranes exhibit much higher flux (~12 times) without any loss of rejection ratio during the separation of water-in-hexadecane emulsion. The significant improvement of separation performance can be attributed to the following issues: Firstly, the existence of wrinkles results in higher surface roughness, providing an additional driving force for separation resulting from the enlarged contact-angle difference between water and oil; Secondly, the shrinkage of the supporting PLLA layer during recovery induces the preferred alignment of CNTs along the wrinkle direction, which is the reason for the orientated slit pores with enhanced overlap of neighboring pores in the film-thickness direction; Finally, a wrinkled surface significantly increases the available area for separation. The synergism of the effects discussed above contributes to much higher permeability and comparable selectivity relative to the reference.

## 1. Introduction

Separation membranes have been widely used in various fields. During separation, two parameters, including rejection ratio and flux, were introduced to assess selectivity and permeability, respectively. The former can be improved by decreasing pore size. Smaller pores, however, lead to the loss of the latter, and vice versa. This is the well-known trade-off effect in separation [1,2,3,4]. So far, much effort has been made to break this effect. Firstly, various strategies have been introduced to modify wettability of the adopted liquid. For instance, poly(ethylene terephthalate) (PET) track-etched membranes have been widely used in membrane distillation. Its hydrophobic property, therefore, is its key as a barrier to liquid and as a medium for vapor transfer [5,6,7]. To fabricate hydrophobic PET membranes, Korolkov et al. developed a novel strategy via the immobilization of hydrophobic nanoparticles. In the attained membranes, the permeability increased remarkably with comparable selectivity [8,9]. Secondly, pore geometry has received much attention since it plays an important role in separation. According to the simulation result from Brans, membranes with slit pores showed much higher permeability and selectivity relative to circular pores [10]. This conclusion has been validated by Yang et al. [11], who prepared polycaprolactone (PCL) membranes via electrospinning. The anisotropic separation membranes with improved permeability and selectivity were obtained via uniaxial tension at different deformation levels. Finally, it was demonstrated that a wrinkled membrane is a promising solution to enhance separation performance, in which the available area for permeation can be improved remarkably. Jin et al. fabricated crumpled polyamide/carbon-nanotube (PA/CNTs) composite membrane by taking nanoparticles as a sacrificial template [12]. The resultant nanofiltration membrane exhibited simultaneous improvement of flux and rejection ratio during desalination. In the fabrication of nitrile butadiene rubber latex/graphene oxide (NBR/GO) membranes, Munusamy et al. also observed the formation of ridges and grooves. The improved surface area for filtration contributed to much higher flux [13]. Therefore, wrinkled membranes with special wettability, orientated pores, and increased separation area can contribute to much better separation performance based on the combination of the effects discussed above. Their fabrication, however, always concerns complicated processes (e.g., interfacial polymerization and sacrificial templating material, as well as the removal of it). A facile strategy for this purpose is highly desired but remains challenging.

Shape-memory polymers (SMPs) are functional materials that can fix their temporary shape in certain conditions and recover to the permanent shape upon external stimuli [14,15,16]. As a result, SMPs have been adopted as the substrate to prepare wrinkled structures. For instance, Xie et al. reported an efficient approach to fabricate wrinkles of metals. In a typical process, thin metal film was deposited onto the surface of pre-deformed shape-memory polymer, followed by SMP being released at high temperature [17,18]. The compressive strain of the deposited metal film accounts for the occurrence of wrinkles. In the results from Xiao and Wang [19], the wrinkled surface was prepared with the help of the deformation and recovery of polymer/polymer bilayers. The resultant period and amplitude of the wrinkles exhibit crucial dependence on film thickness, strain of SMPs, etc. Therefore, there have been some reports concerning wrinkles based on shape-memory polymers [20,21,22,23,24,25]. Most of them, however, focus on the compact films. Little attention has been paid to the fabrication of separation membranes with wrinkled surfaces based on shape memory effect, which can be attributed to the absence of shape-memory-supporting membranes with both excellent mechanical performance and interpenetrated separation channels [26,27].

**Scheme 1:**
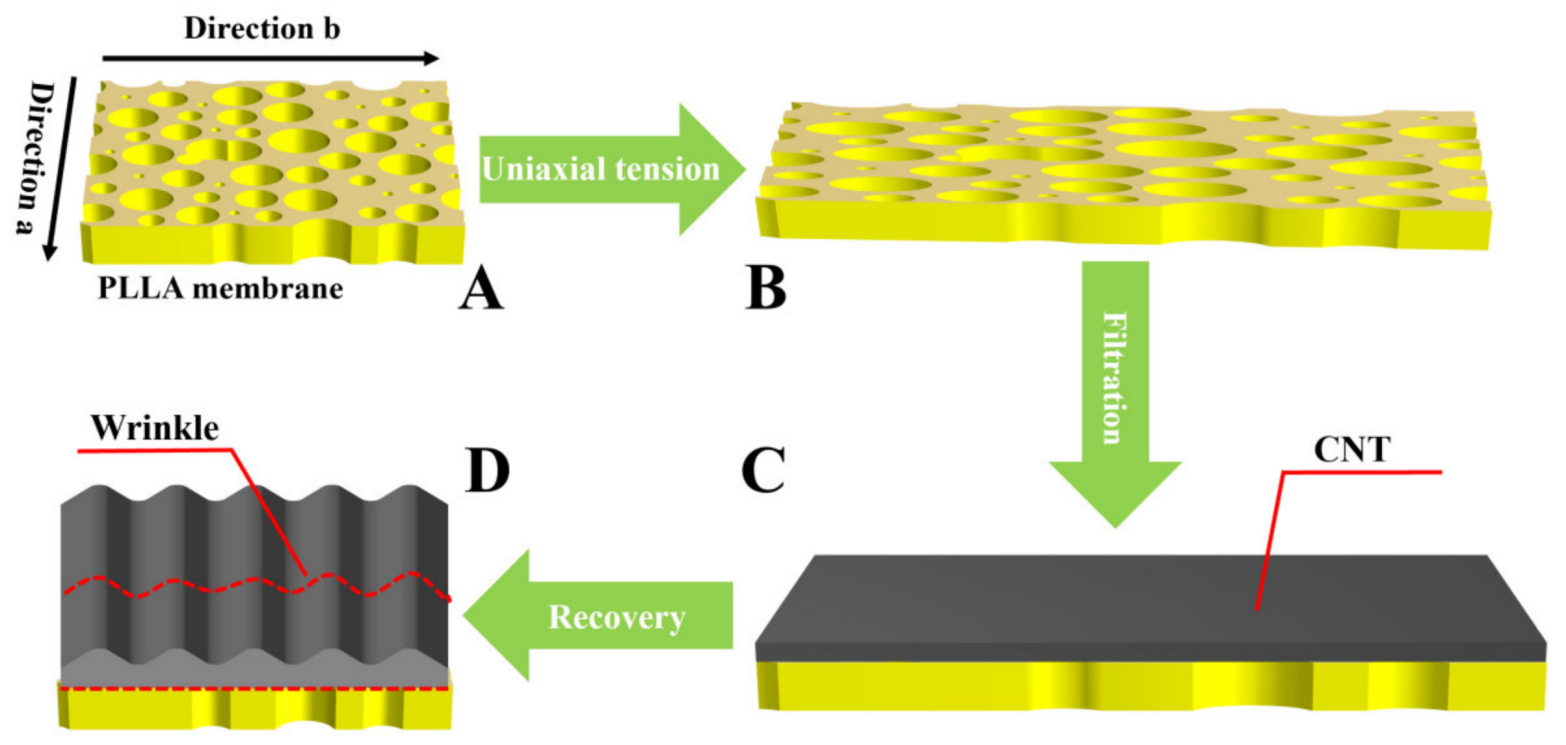
Fabrication of CNT separation membranes with wrinkles based on shape memory effect of PLLA (**A**) via uniaxial tension (**B**), loading of CNTs (**C**) and formation of membranes (**D**).

In our previous work, porous PLLA membranes were fabricated by spin-coating (or blade-coating) poly(L-lactic acid)/polyethylene oxide (PLLA/PEO) blend solution and the subsequent removal of PEO by water etching [26]. The attained PLLA membranes exhibited interpenetrated diffusion channels. PLLA, a typical shape-memory polymer with excellent mechanical performance and suitable switching temperature, can act as the supporting layer to prepare wrinkled membranes with the help of its recovery [28,29,30]. On the other hand, CNTs are perfect candidates for the top (i.e., wrinkled) layer due to their relatively rigid performance and smaller size [31]. In this work, therefore, it is proposed to fabricate wrinkled CNT membranes on porous shape-memory PLLA membranes (Scheme 1A). On the pre-deformed PLLA membranes (Scheme 1B), CNTs can be filtered from their suspensions (Scheme 1C). Upon heating, PLLA membranes tend to shrink while the CNT layer remains stable. The asynchronous deformations of CNTs and PLLA layers produce aligned surface wrinkles (Scheme 1D). The wrinkled surface of CNTs@PLLA composite membranes is beneficial for the improvement of separation performance, according to the following issues: Firstly, the enhanced surface roughness results in special wettability, providing an additional driving force for separation; Secondly, the preferred CNT alignment accounts for the orientated slit pores. The resultant pore geometry can improve permeability. Finally, surface wrinkles lead to a larger available area and higher flux. The synergism of them can contribute to the significant improvement of separation performance. Therefore, CNTs@PLLA composite membranes have been prepared successfully and used in the separation of water-in-oil emulsion. Relative to reference without wrinkles, our specimen exhibits much better separation performance.

## 2. Materials and Methods

The single-walled carbon nanotubes (SWCNTs) were provided by Xuzhou Jiechuang Materials Technology Co., Ltd. (Xuzhou, China). Polylactic acid (PLLA, 4032D) was purchased from Nature Works (Minneapolis, MN, USA). Polyethylene oxide (PEO) was purchased from Sigma-Aldrich Trade Co., Ltd. (Shanghai, China). Chloroform, Tween 80 and Span 80 were supplied by Shanghai Lingfeng Chemical Reagent Co., Ltd. (Shanghai, China). Acetone was achieved from Hangzhou Shuanglin Chemical Reagent Co., Ltd. (Hangzhou, China).

The CNT/water suspension was prepared by mixing CNTs, deionized water, acetone, and Tween 80 with weight ratio of X (X = 1.0, 1.5, 2.0, 2.5)/80/20/0.5 at room temperature and sufficient ultrasonic treatment. Porous shape-memory PLLA membranes have been fabricated by blade-coating PLLA/PEO blend solution (in chloroform with concentration of 4 wt%) with the weight ratio of 1:1. After evaporation of chloroform, the specimen was immersed in deionized water to remove PEO. The free-standing porous PLLA membranes can then be obtained according to the preparation methods discussed in reference [26]. Uniaxial tension was achieved in a water bath of 63 °C, followed by cooling down to room temperature with deformation. The CNTs@PLLA composite membrane was prepared by filtrating (at negative pressure of 0.02 MPa, setup shown in Figure S1) the CNTs’ suspension. Then, the attained composite membrane was kept in 1 L water for 48 h and then in vacuum for another 48 h, followed by hot-pressing at 40 °C and 15 MPa for 5 min. Subsequently, the composite membrane was restored in a water bath at 63 °C for the recovery of PLLA.

The surface/fracture morphologies of CNTs@PLLA composite membranes were observed by scanning electron microscopy (SEM, Hitachi S-4800, Tokyo, Japan). The contact angles were measured on Drop Shape Analysis (DSA 100, Krüss, Hamburg, Germany) with a droplet of 3 μL. The 3-D morphology of composite membrane was characterized by digital microscope (DSX 1000). The electric resistance measurement was characterized by an ultrahigh-resistivity meter with a URS probe electrode (model MCP-HT450) at atmospheric temperature. The measurements of oil flux were performed using a dead-end filtration at negative pressure of 0.1 MPa. The volume of tested emulsion in the experiment was 10 mL. To assess the permeability stability of the prepared membranes, the flux of oil/water emulsion was measured with volume of 1000 mL. The flux was calculated according to Equation (1):

(1) $$F=\frac{V}{A\times t\times P}$$

where *V*, *A*, *t*, and *P* are total volume of permeated emulsion, membrane area, operation time, and adopted pressure, respectively. The rejection ratio was measured with water-in-hexadecane mixtures. The surfactant-stabilized emulsion was prepared by mixing deionized water, hexadecane, and Span 80 with the weight ratio of 50/1//0.01 and stirring for 3 h at 1400 rpm. The rejection, R, was calculated according to Equation (2):

(2) $$R=\left(1-\frac{C_{p}}{C_{f}}\right)\cdot 100\%$$

where *C_f_* and *C_p_* are water content of feed emulsions and filtrate, respectively. The water concentration was measured by Karl Fisher titration (Metrohm 831 KF Coulometer, Herisau, Switzerland). Optical Microscope (DP72, Olympus, Tokyo, Japan) was used to observe water droplets and their size distribution in oil before and after filtration.

## 3. Results and Discussion

**Figure 1:**
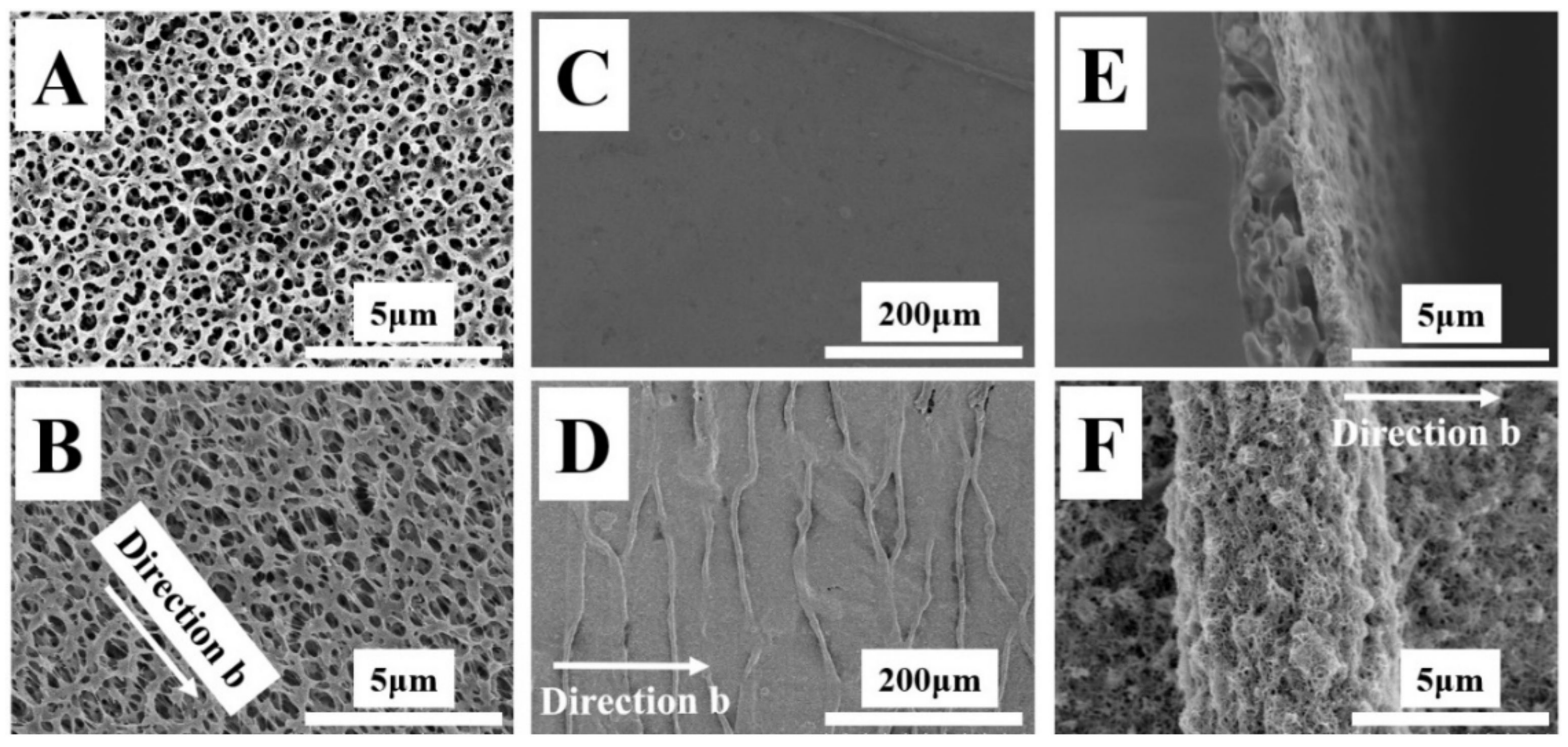
SEM images of porous PLLA membranes (**A**), deformed membranes (**B**), CNTs loaded on PLLA membranes (**C**) and membranes with wrinkles after recovery of PLLA (**D**). (**F**) shows one wrinkle with higher magnification and (**E**) illustrates the cross-section of the composite membrane of CNTs@PLLA.

PLLA is a typical shape-memory polymer, in which tiny crystals resulting from low crystallinity and amorphous matrix serve as the shape-fixed phase and shape-recovery phase, respectively [27,28,29,30]. Shape memory effect plays an important role in the fabrication of composite membranes with wrinkled surfaces based on their deformation and recovery. As shown in Figure 1A, PLLA membranes with round pores overlapping with each other can be prepared via blade-coating, according to the strategy developed in our previous work [26]; this is its permanent shape. Uniaxial tension (draw ratio = 1.7) at high temperature (65 °C, here) produces elliptical pores with a length/width ratio of ~1.7, indicating a proportional deformation at macroscopic and microscopic scales. This is the temporary shape of the PLLA membrane, which can be fixed by cooling down to room temperature under strain. It is facile to fabricate CNTs@PLLA composite membranes by filtrating the CNTs’ suspension at the pre-deformed PLLA membranes in their temporary shape. As shown in Figure 1C, a flat layer of CNTs can be obtained after filtration. The thicknesses of the CNT layer and supporting PLLA layer are ~550 nm and ~1800 nm, respectively (Figure 1E). When the composite specimen is heated above the switching temperature of PLLA, i.e., the glass transition temperature (~61 °C, determined by means of DMA, Figure S2), PLLA tends to recover to its permanent shape shown in Figure 1A, while the CNT layer does not exhibit any deformation ability. As a result of asynchronous deformation, CNTs’ wrinkles can be obtained on top of the PLLA supporting layer (Figure 1D). In SEM images with higher magnification (Figure 1F), there are ridges and valleys, both of which are composed of CNTs. The width of the former is roughly 5 μm. Obviously, the structure of wrinkles depends crucially on preparation conditions, including CNT content in suspension, volume, and draw ratio during uniaxial tension. The details can be found in the supporting information (Figures S3–S5).

**Figure 2:**
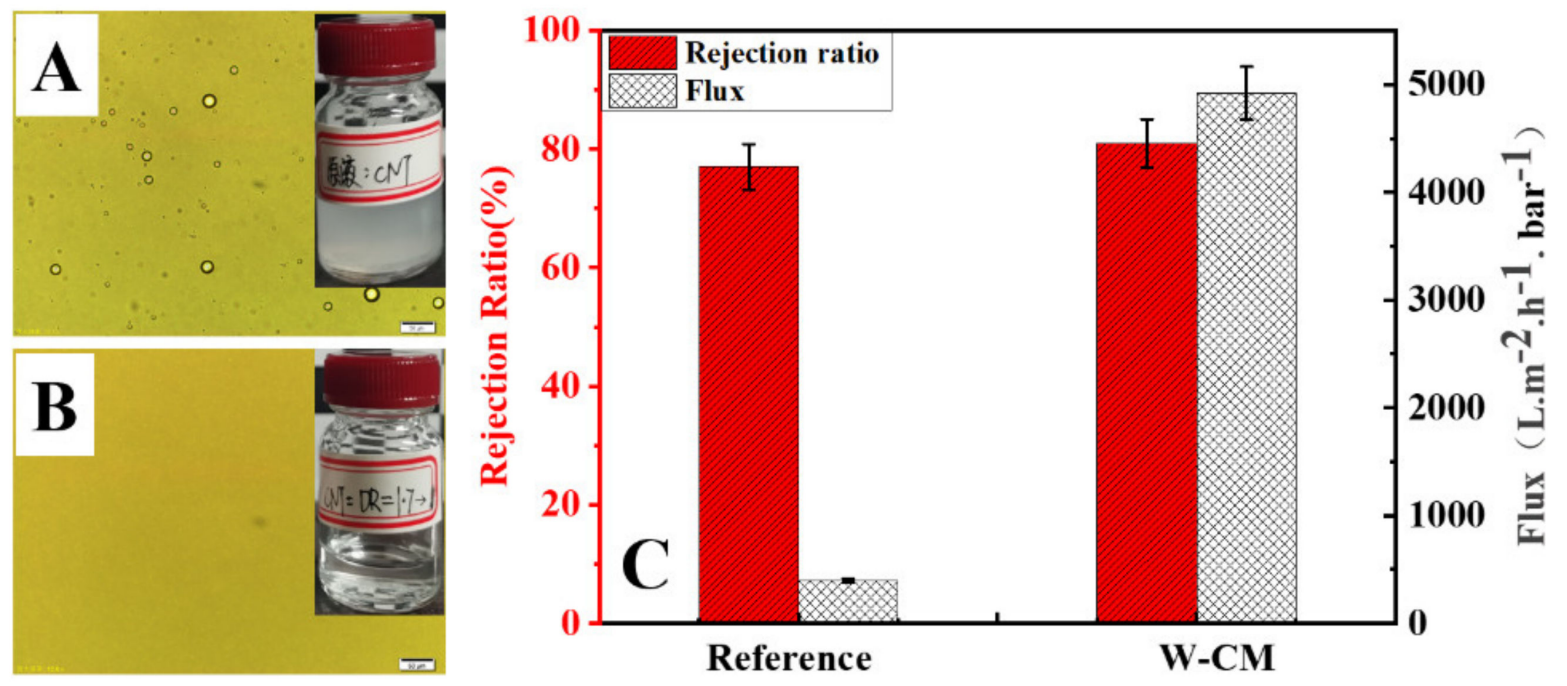
POM images of water-in-oil emulsion before (**A**) and after separation (**B**). (**C**) shows the flux and rejection ratio during the separation of water-in-oil emulsion. Insets in (**A**,**B**) are the corresponding photography.

The attained CNTs@PLLA composite membrane exhibits excellent mechanical performance (with the stress of 2–3 MPa and elongation at break of ~40%, reference [26]) and hydrophobic/oleophobic properties (data will be shown in the following sections). As a result, it can act as a promising candidate for the separation of water-in-oil emulsion. In this work, the emulsion was prepared by stirring hexadecane/water/Span 80 (with a weight ratio of 50/1/0.01) at 1400 rpm for 3 h. As shown in the inset of Figure 2A, an opaque emulsion was obtained. In the polarizing optical microscope (POM) image of this emulsion, there are water droplets with diameters ranging from 0.5 μm to 15 μm. Based on the homemade setup shown in Figure S6, the separation of water-in-hexadecane emulsion was carried out with the help of the CNTs@PLLA composite membranes. After the separation with the membrane (with or without wrinkles), there was no water droplet in the POM image (Figure 2B), corresponding to the transparent filtrate. To assess the separation performance of CNTs@PLLA membranes quantitatively, both the flux and rejection ratio during the separation of water-in-hexadecane were tested. During this process, the separation was driven at a negative pressure of 0.02 MPa. Two specimens were adopted, i.e., CNTs@PLLA composite membranes without microwrinkles (before recovery, named as reference) and with microwrinkles (after recovery, named as wrinkled composite membranes, W-CM). As shown in Figure 2C, two specimens exhibit comparable rejection ratios (~80%). The lower magnitude of rejection ratio should be associated with the broad distribution of water-droplet diameters due to the lower content of surfactant (Span 80 here). Their fluxes, however, show notable differences. In the case of reference, the flux is ~400 L × m^−2^ × h^−1^ × bar^−1^. This value increased by ~12 times in the result of W-CM, reaching 4900 L × m^−2^ × h^−1^ × bar^−1^. Therefore, the existence of wrinkles on the composite membranes’ surface contributes to much higher permeability without any loss of selectivity, which can be attributed to the wettability difference between water and oil, the orientation of CNTs, and the increase in separation area. All these factors will be discussed one by one in the following sections. Furthermore, the water-in-oil emulsion with much higher volume (1000 mL) was taken to assess the permeability stability of the composite membranes with wrinkles. According to Equation (1), the average flux is ~4300 L × m^−2^ × h^−1^ × bar^−1^. The slightly decreased flux indicates the excellent anti-fouling performance of CNTs@PLLA composite membranes.

**Figure 3:**
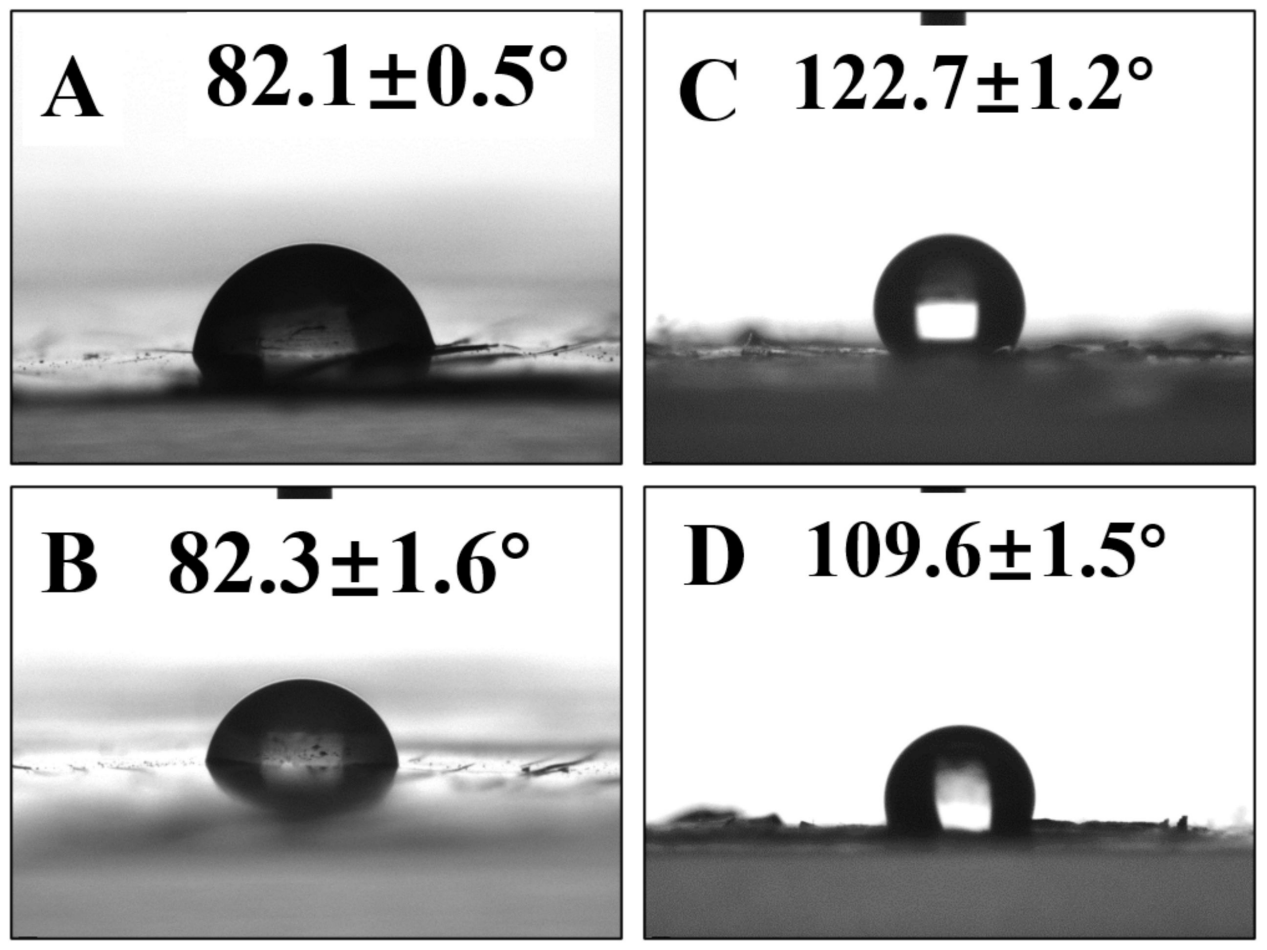
Water contact angles of reference (**A**,**B**) and W-CM (**C**,**D**), observed from Direction a (**A**,**C**) and b (**B**,**D**), respectively.

It is well-known that wettability of water and oil, especially the difference between them, plays an important role in the separation of them. The enlarged contact-angle difference contributes an additional driving force for the separation. In this work, both CNTs@PLLA membranes (reference and W-CM) exhibit super-oleophilic properties. The hexadecane contact angles are close to 0°, corresponding to rapid spreading and absorption of its droplets on membrane surface (Videos S1 and S2). Furthermore, in the case of W-CM, it takes a short time (7 s, Video S2) for hexadecane to enter the composite membrane relative to the reference (14 s, Video S1), indicating better wettability of hexadecane on W-CM. The water contact angles (WCAs) on composite membranes are measured and shown in Figure 3. In reference, the magnitude of WCA is located at ~82° in two directions (Direction a and b). The similar WCAs can be ascribed to the isotropic surface shown in Figure 1C. After recovery at high temperature (Figure 1D), the water contact angles of W-CM are quite different. On one hand, wrinkles along Direction a account for the different water contact angle in two directions (Figure 3B,D). Relative to Figure 3D, the contact angle in Direction a exhibits a higher magnitude (Figure 3B), which can be attributed to the alignment of wrinkles in the corresponding direction. On the other hand, the water contact angles in both directions are much higher than the results before recovery, i.e., the surface of the reference. This result has good agreement with the enhanced surface roughness shown in Figure 1D. As the result of the higher water contact angle, the wettability difference between water and oil (CA = 0°, shown in Video S1) improved. This is the reason for the additional driving force during the separation, and the enhanced separation performance.

**Figure 4:**
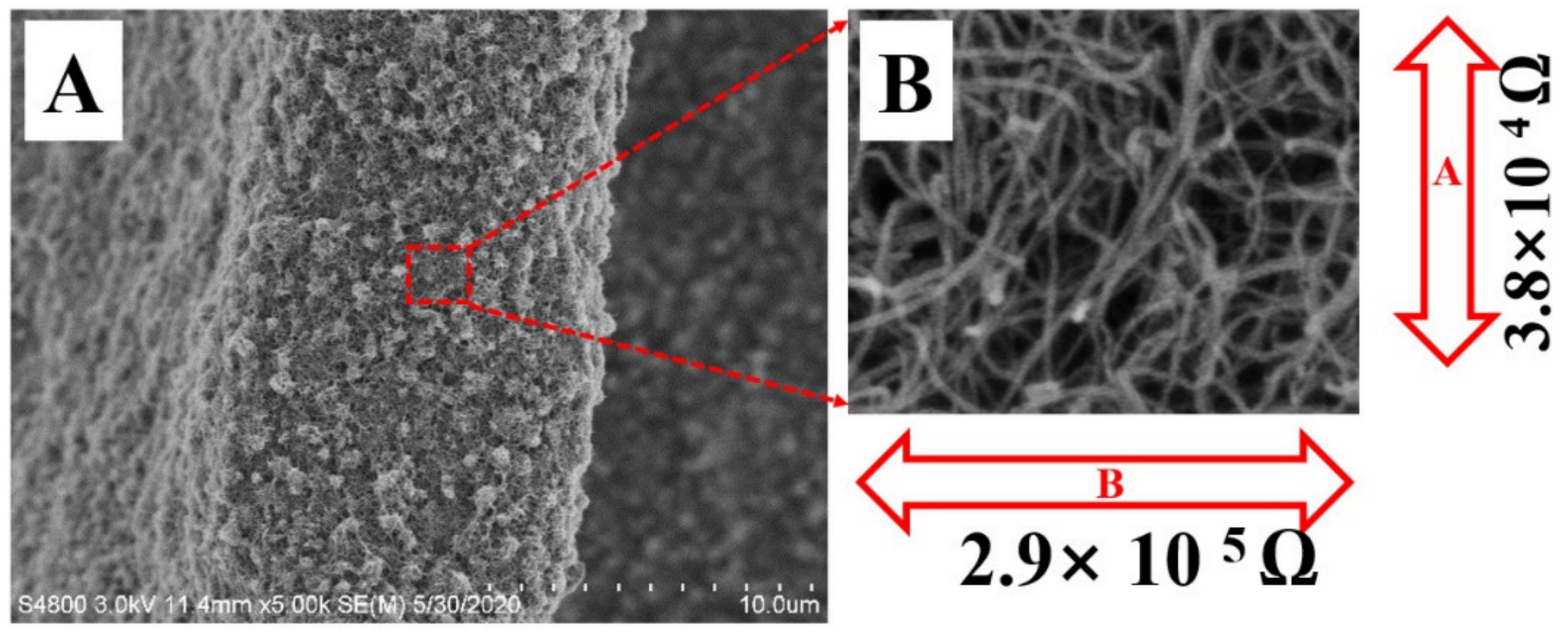
SEM images of one certain wrinkle (**A**) and the corresponding image with higher magnification (**B**) to show the preferred alignment of CNTs along direction A. The numbers in (**B**) represent the magnitude of electric resistance in the indicated directions.

The CNTs@PLLA composite membranes are composed of a CNT layer on top and a PLLA layer at the bottom. The pore size of the former exhibits much lower magnitudes relative to the latter. Therefore, the pores among the CNTs determine the permeability and selectivity. There are isotropic pores in the CNT layer in the as-prepared membranes (i.e., reference, Figure 1C). Upon recovery, shrinkage of the PLLA layer along Direction b results in the compressive strain and preferred alignment of CNTs in Direction a. This is the reason for the occurrence of wrinkles (Figure 1D and Figure 4A), higher water contact angles in this direction (Figure 2C), and orientated slit pores. It has been demonstrated that pore geometry plays an important role in water/oil separation. Relative to the circular pores, slit pores exhibit much higher flux and rejection ratio due to their shorter diffusion length and smaller width [32]. To clearly show the alignment of CNTs, our attention focused on the following issues: First, the SEM image with higher magnification was taken and is shown in Figure 4B. There is an obvious orientation of CNTs along Direction a. On the contrary, all CNTs distribute randomly on the surface of the reference (Figure S7). Second, the difference in electric resistance in two directions can be used to describe the preferred alignment of CNTs, since only CNTs are conductive in the composite membranes. As shown in Figure 4B, their magnitudes are 3.8 × 10^4^ Ω and 2.9 × 10^5^ Ω in Direction a and b, respectively. The lower magnitude in Direction a suggests the preferred alignment of them in this direction. According to the discussion above, it is obvious that more CNTs array along Direction a, producing not circular pores, but slit pores. The resultant orientated slit pores account for the enhanced overlap of neighboring pores in film-thickness direction, contributing to the shorter diffusion length and higher flux.

**Figure 5:**
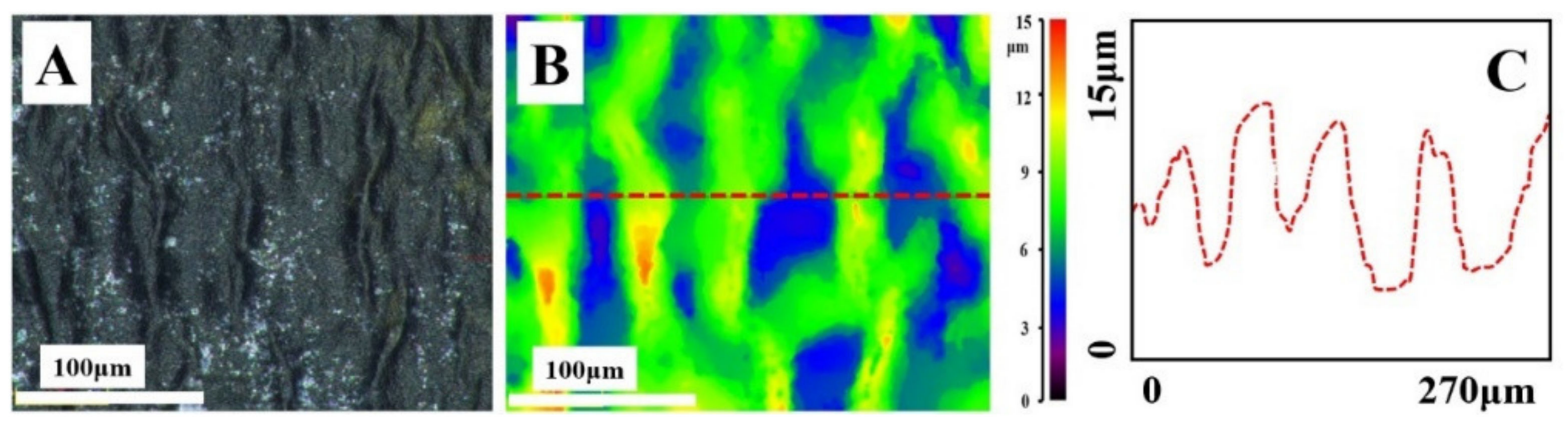
Digital microscope images of CNTs@PLLA composite membranes with wrinkle (**A**), the 3-D morphology (**B**), and corresponding line-cut profile at the indicated position in (**C**).

According to Darcy’s law (Equation (3)) [33,34], the flux shows linear dependence on the separation area.

(3) $$Q=\left(-kA\left(P_{i}-P_{f}\right)\right)/\mu L$$

where *A*, *k*, *P_i_*, *P_f_*, *μ* and *L* are the available testing area, coefficient of permeability, pressure of input, pressure of output, viscosity, and diffusion length of fluid, respectively. Jin et al., fabricated nanofiltration membranes with surface wrinkles [12]. The resultant membrane exhibits better separation performance, which is attributed to the increase in available area. In this work, there are wrinkles on the membrane surface (Figure 1D,F). The consequent separation area can be improved. To show this effect clearly, a digital microscope with various viewing angles was employed to obtain the 3D structures of the composite membranes. As shown in Figure 5A, the wrinkles are obvious, which agrees well with the SEM images (Figure 1). The corresponding 3D image (Figure 5B) presents more details of the ridge and valley of wrinkles. Two parameters were introduced to describe the wrinkles. The period represents the distance among neighboring ridges or valleys, while the amplitude is defined as the height difference between the ridge and valley. From Figure 5A,B, it is obvious that the period is roughly 50–60 μm, which has good agreement with the SEM images (Figure 1D). With the help of 3D images, the height difference between the ridge and valley, i.e., the amplitude of wrinkles was measured. It is located at 8–9 μm. Figure 5C shows the line-cut profile at the indicated position in Figure 5B. This profile makes it clear that the CNTs@PLLA composite membrane exhibits an extremely rough surface with an amplitude of several microns. The root-mean-square (RMS) roughness is 2.4 microns. The enhanced roughness not only enlarges the water contact angles based on the Cassie effect, but also induces the anisotropic surface and different water contact angles in two directions (Figure 3B,D). Furthermore, it is facile to calculate the area ratio between the wrinkled surface and flat surface according to this profile [12,35,36]. Its value is ~2.7, suggesting that the available separation area has increased significantly. The higher magnitude of separation area contributes to higher flux during separation. The digital-microscope image of the reference is not shown here since there are no obvious wrinkles, which is in good agreement with the SEM image (Figure 1C).

According to the discussion above, we can describe the formation of the surface CNTs’ wrinkles, and their role during the improvement of separation performance, as follows: The temporary shape of PLLA membranes upon uniaxial tension is fixed by cooling to room temperature (Figure 1A). CNTs can be loaded on top of PLLA layer by filtrating its suspension. Upon heating to above the switching temperature (i.e., glass transition temperature of PLLA), the PLLA membranes tend to recover to their permanent shapes, resulting in the significant shrinkage of the supporting layer in Direction b. The top CNT layer, however, does not exhibit any deformation ability. The asynchronous deformation of the top CNT layer and bottom PLLA layer produces a compressive strain of CNTs, accounting for the wrinkled separation layer along Direction a. The resultant composite membranes with wrinkled surfaces influence the separation of water-in-hexadecane emulsion based on special wettability (from enhanced surface roughness), orientated slit pores (from preferred CNT alignment), and higher available area for separation. Firstly, wrinkles with ridges and valleys enhance the surface roughness. This is the reason for the enlarged contact-angle difference between water and hexadecane, accounting for the additional driving force during separation of their emulsion (Figure 3). Secondly, the available separation area of the composite membranes increased remarkably due to the existence of wrinkles and a rough surface, which was demonstrated in Figure 5B,C. Finally, the shrinkage of the PLLA membrane induces the preferred alignment of CNTs along Direction a, resulting in slit pores instead of circular pores (Figure 4). The slit pores correspond to the enhanced overlap of neighboring pores in the film-thickness direction, accounting for the shorter diffusion length. During the separation of water-in-hexadecane emulsion, the synergism of the effects discussed above contributes to much higher permeability and comparable selectivity relative to the reference without wrinkles.

## 4. Conclusions

Based on the recovery of PLLA membranes with shape memory effect, wrinkled CNTs@PLLA composite membranes with an amplitude of several microns and a period of tens of microns have been fabricated. The attained surface wrinkles contribute to higher surface roughness and available area of separation membrane. The former is the reason for the enlarged contact-angle difference between water/hexadecane and the additional driving force during separation. The latter accounts for the higher flux according to Darcy’s law. During the formation of wrinkled membranes, the shrinkage of the supporting PLLA layer induces the preferred alignment of CNTs along the direction of wrinkles, resulting in slit pores and the enhanced overlap of neighboring pores in film-thickness direction. In the separation of water-in-oil emulsion, relative to the reference without wrinkles, the synergism of enlarged contact-angle difference, increased separation area, and the alignment of CNTs produces much higher flux (~12 times) without any loss of rejection ratio. Our result provides a novel solution for trade-off effect between permeability and selectivity in separation.

## Data Availability

Not applicable.

## Supplementary Materials

The following supporting information can be downloaded at: https://www.mdpi.com/article/10.3390/membranes12030278/s1: Figure S1: Home-made setup for the loading of CNTs on PLLA membranes by filtrating CNTs suspension, Figure S2: DMA (Tanδ) curve of obtained porous PLLA, Figure S3: SEM images of CNTs loaded on PLLA membranes and wrinkled membranes with different draw ratios (1.0 (a), 1.5 (b), 1.7 (c), 2.0 (d)) after recovery of PLLA, Figure S4: SEM images of wrinkled membranes (Draw ratio = 1.7) with different volume (1.5 mL (Ⅰ), 2.0 mL (Ⅱ), 2.5 mL (Ⅲ), 3.0 mL (Ⅳ)) after recovery of PLLA, Figure S5: SEM images of wrinkled membranes (Draw ratio = 1.7, Volume = 2.5 mL) with different CNT contents in suspension (0.01 mg mL^−1^ (A), 0.015 mg mL^−1^ (B), 0.02 mg mL^−1^ (C), 0.025 mg mL^−1^ (D)) after recovery of PLLA, Figure S6: Image of homemade separation device, Figure S7: SEM images of reference with low (A) and high (B) magnifications, Video S1: Contact angle evolution on reference, Video S2: Contact angle evolution on W-CM.
