# Supplementary material for: Wrinkled CNTs@PLLA Composite Membranes for Enhanced Separation Performance
Source: Membranes (Basel). 2022 Feb 28;12(3):278. doi: 10.3390/membranes12030278 (PMC8948802; doi:10.3390/membranes12030278)
Supplement: Supplementary file 1 [file membranes-12-00278-s001.zip › Supporting Information.pdf]

# Supporting Information

# Wrinkled CNTs@PLLA Composite Membranes for Enhanced Separation Performance

Jinyan Xu[1], Bajin Chen[2], Lu Yin[1], Liang Zhang[1], Yongjin Li[1], Jichun You[1,*]

1. *College of Material, Chemistry and Chemical Engineering, Key Laboratory of Organosilicon Chemistry and Material Technology, Ministry of Education, Hangzhou Normal University, Hangzhou, 311121, Zhejiang, China; xjy5282022@163.com (X, J) 15967102282@163.com (Y, L) layzhang@foxmail.com (Z. L), yongjin-li@hznu.edu.cn (L. Y)*
2. *Transfar Zhilian Co. Ltd, Xiaoshang, Hangzhou, 311215, China; 4437@etransfar.com (C. B)*

*Corresponding author, Prof. You J. you@hznu.edu.cn

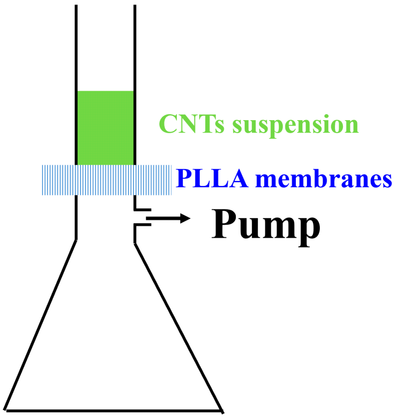


Figure S1, Home-made setup for the loading of CNTs on PLLA membranes by filtrating CNTs suspension.

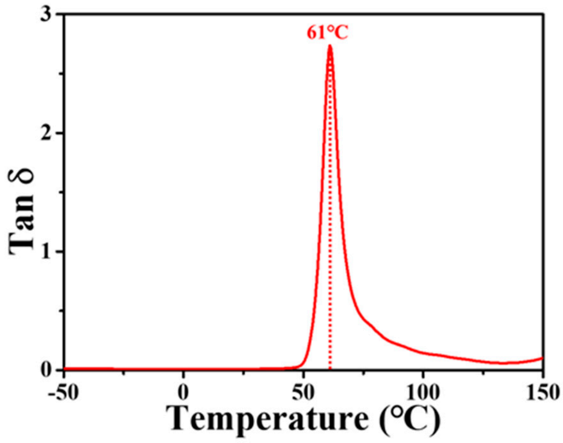


Figure S2, DMA (Tanδ) curve of obtained porous PLLA.

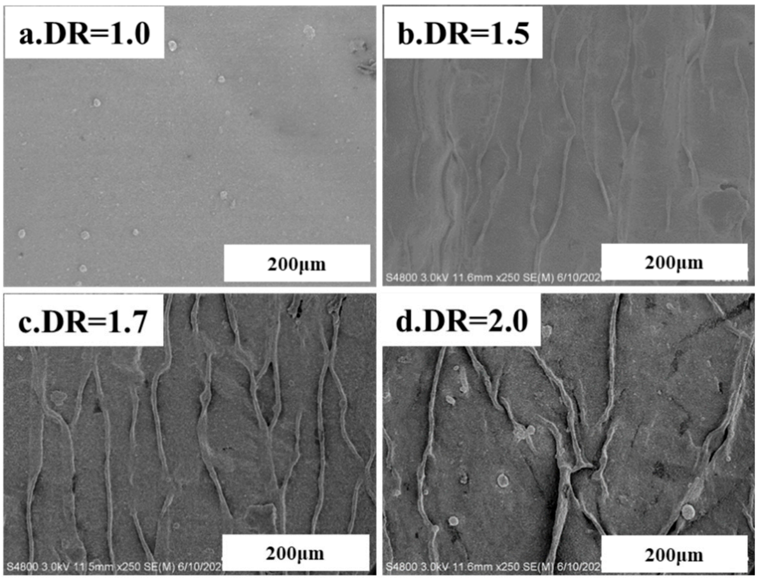


Figure S3, SEM images of CNTs loaded on PLLA membranes and wrinkled membranes with different draw ratios (1.0 (a),1.5 (b),1.7(c),2.0 (d)) after recovery of PLLA.

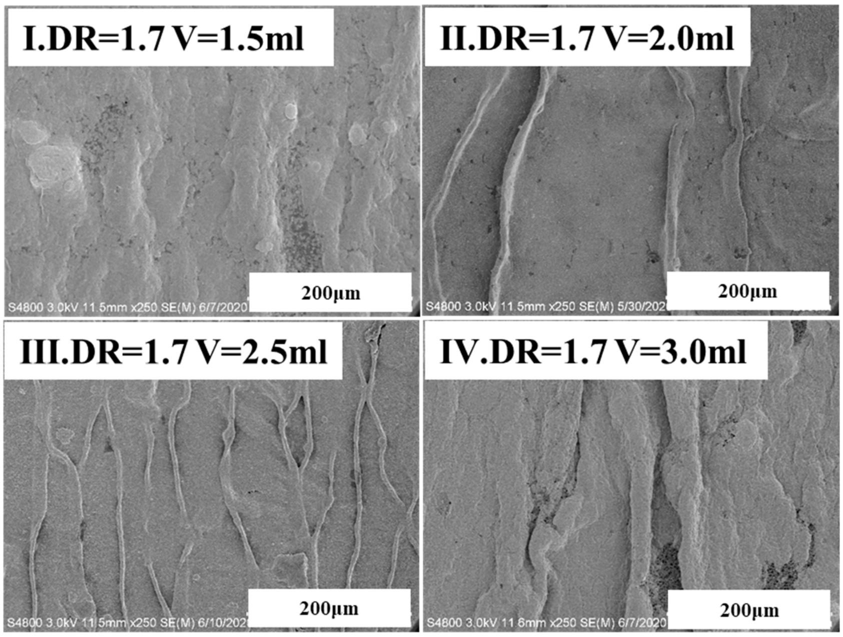


Figure S4, SEM images of wrinkled membranes (Draw ratio=1.7) with different volume (1.5ml(I),2.0ml (II),2.5ml (III),3.0ml (IV)) after recovery of PLLA.

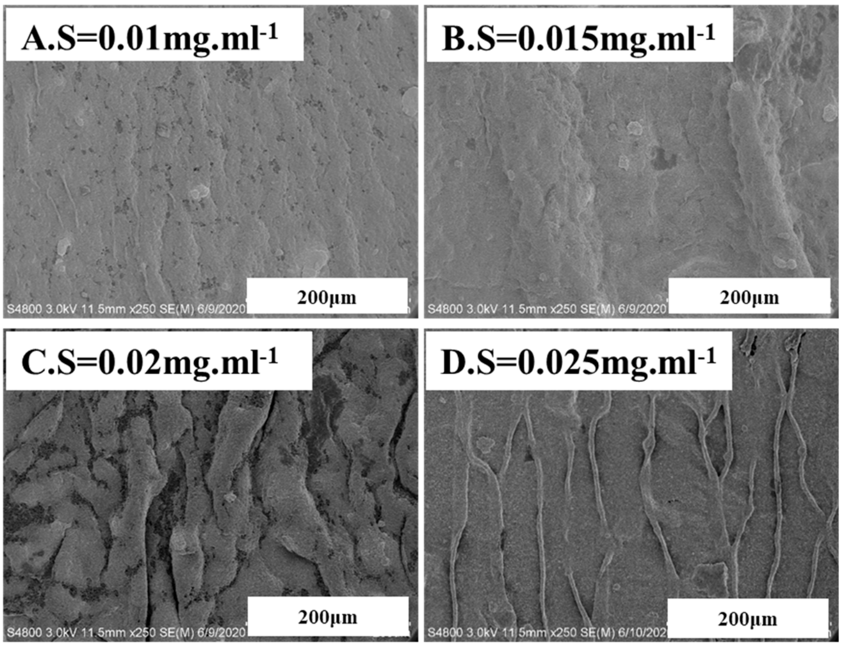


Figure S5, SEM images of wrinkled membranes (Draw ratio=1.7, Volume=2.5ml) with different CNTs contents in suspension (0.01mg.ml$^{-1}$ (A),0.015 mg.ml$^{-1}$ (B),0.02 mg.ml$^{-1}$ (C),0.025 mg.ml$^{-1}$ (D)) after recovery of PLLA.

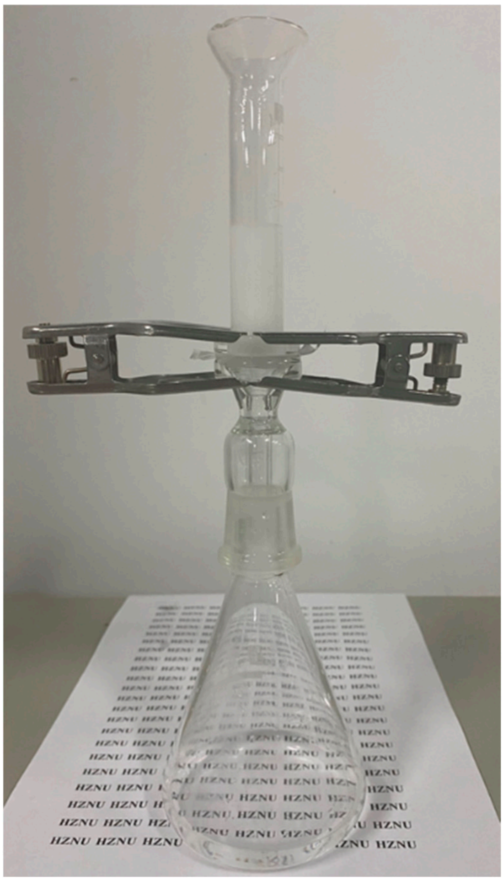


Figure S6, Image of home-made separation device.

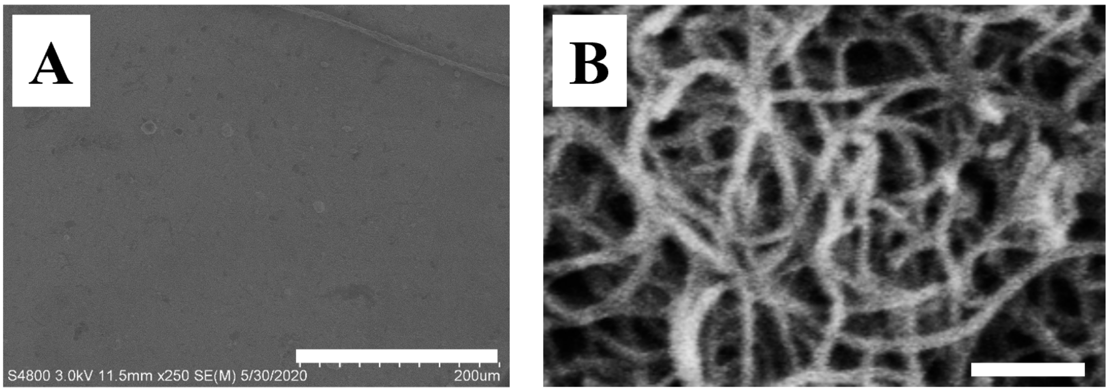


Figure S7, SEM images of reference with low (A) and high (B) magnifications. The scale bars in (A) and (B) are 200 microns and 200nm respectively.
